# The time is ripe for ESG + Nutrition: evidence-based nutrition metrics for Environmental, Social, and Governance (ESG) investing

**DOI:** 10.1038/s41430-022-01075-9

**Published:** 2022-01-24

**Authors:** Meghan O’Hearn, Suzannah Gerber, Sylara Marie Cruz, Dariush Mozaffarian

**Affiliations:** 1 Friedman School of Nutrition Science and Policy, Tufts University, Boston, MA USA; 2 Tufts Medical Center, Tufts University School of Medicine, Boston, MA USA

**Keywords:** Risk factors, Obesity, Metabolic syndrome

The globe faces a nutrition crisis. Suboptimal diet is the leading cause of poor health worldwide, with devastating social, environmental, equity, and economic consequences [1]. In 2018, poor diet quality was estimated to cause 12 million deaths due to non-communicable diseases (NCDs) globally [1]. In the US, treatment of cardiovascular diseases, diabetes, and cancers accounted for 1 in 4 dollars in healthcare—and 18% higher spending than in 2009 [2]. Solutions to address the global health and economic burdens of nutrition-related disease must re-imagine and reform the food system – including new approaches to influence the private sector, which plays a critical role in supplying and influencing food choices, nutrition, and health outcomes of consumers.

Among different levers, investors – including institutional investors, family offices, and venture capital – are powerful and underutilized stakeholders for stimulating change. The rise of Environmental, Social, and Governance (ESG) investing presents a remarkable new opportunity to align financial returns with benefits for society and the planet. This paradigm shift recognizes that long-term financial performance is directly linked to environmental and societal impact [3]. From 2012 to 2020, the value of global ESG-driven assets tripled to $40.5 trillion [4], and now represents nearly half of the world’s financial assets under management. Businesses have taken note. In 2021, 60 top global businesses committed to publicly supporting and reporting on a common set of Stakeholder Capitalism Metrics for ESG reporting [5]. And, at the 2021 UN Climate Change Conference (COP26), The International Financial Reporting Standards (IFRS) Foundation announced a new International Sustainability Standards Board to develop, consolidate, and govern sustainability disclosure standards for businesses [6].

However, ESG metrics to-date have largely highlighted the Environmental and Governance domains, with little to no Social-focused metrics (and mostly related to employees), and virtually none for nutrition and health [7, 8]. Given the major impact of the food sector on well-being, it’s imperative that new ESG metrics be developed to guide investors to prioritize businesses that innovate responsible practices aligned with consumer health and to divest from those who do not (Fig. 1).

**Fig. 1:**
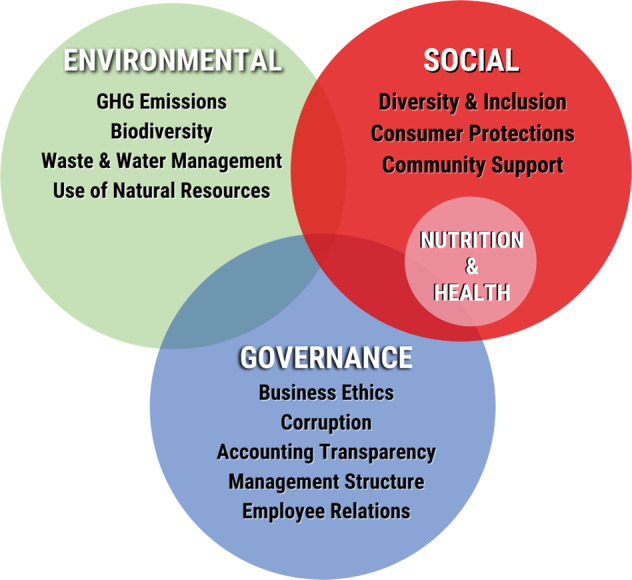
The Environmental, Social, and Governance (ESG) investing framework: a need to add nutrition and health. Core elements included within each of the Environmental, Social, and Governance domains, highlighting the importance of nutrition and health within the Social domain.

To be successful, such ESG + Nutrition metrics must be measurable, evidence-based, accurately reflect benefits and harms on consumer nutrition and health, and track with long-term financial performance. We believe the business case is clear. We also believe the alignment of investment decisions with consumer nutrition and health could create as large a public health impact as global efforts around consumer education and government food policy.

## ESG + Nutrition: addressing investment risks of the food sector

The growing evidence and recognition of the impact of suboptimal nutrition on chronic diseases present multiple risks for the long-term reputation, viability, and fiscal performance of food sector businesses [9]. For example, regulatory risks for food sector businesses to address nutrition are rapidly growing, such as national front-of-package labels, warning labels, taxation, procurement policies, limitations on additives, and marketing restrictions [10, 11]. Reputational risks are similarly mounting, through societal pressure to hold private sector actors accountable for their role in diet-related health and advocacy efforts to expose tactics used by food sector businesses that harm public health [12]. Market forces mirror these trends, as many consumers, in particular younger consumers, are demanding healthier, more authentic food and beverage products as well as greater transparency around these priorities [12–14]. The negative externalities of poor nutrition and associated disease burdens, including lost work productivity and increased healthcare spending, likewise present major financial risks for not only the food sector, but all private sector enterprises and national economies [15]. At the same time, the UN Sustainable Development Goals (SDGs) are serving as a compass to steer investment frameworks, with food and nutrition central to many of the SDGs [16]. These trends, together with accelerating demand for stakeholder-centric business models focused on long-term value creation for all relevant parties [17], create tremendous risks for investing in the food sector. The corollary of these risks is the tremendous opportunity they provide for driving financial success through the development and distribution of food products that improve health, increase health equity, and reduce healthcare spending.

## A need for objective, measurable, evidence-based ESG + Nutrition metrics

Among major existing ESG frameworks, several have begun to consider consumer nutrition and/or health (Table 1). However, most emphasize company commitments rather than actions; have varying (and often not nutrition-focused) scope; have ambiguous requirements on data collection and analysis; or have not connected the proposed metric with health or financial materiality. For example, the World Benchmarking Alliance (WBA) framework almost exclusively evaluates a company’s own commitments and targets [18], rather than a company’s product portfolio, sales, marketing, and community engagement, which more directly influence consumer nutrition and health. The Global Reporting Initiative (GRI) and Sustainability Accounting Standards Board (SASB) incorporate some more quantitative, nutrition-oriented metrics [19, 20], but with superficial, incomplete determinants of product healthfulness (e.g., sales volume of products that are lowered in saturated fat, trans fat, sodium, and added sugar) [19]. Other proposed metrics are similarly narrow in scope, e.g., “sales growth of fruit and vegetables” (Access to Nutrition Index [ATNI] UK Retail Spotlight) [21]. Other measures are subjective; for example, SASB’s restaurant framework includes the “percentage of meal options consistent with the national dietary guidelines” [20] – without clear methodology for defining a meal option or assessing adherence to national guidelines. Some metrics contain circular definitions, without specification of how impacts should or could be assessed; e.g., the Embankment Project for Inclusive Capitalism lists the “number of people with improved quality of health through sales of products and services” [22]. The ATNI has the most comprehensive (100+) array of metrics for evaluating large multinational food and beverage manufacturers as well as UK retailers, covering product healthfulness, marketing to children, and company strategy [21]. Most of ATNI’s metrics are categorical (thereby requiring less precise data), but may create reporting fatigue and do not cover major food sectors like restaurants or other food services.

**Table 1:** Major existing ESG frameworks and standards relating to the food sector.

ESG framework, standard or assessment^a^	Sector Focus^b^
Global Reporting Initiative: GRI food processing sector supplement [19]	Generic
Food processing	Generic
World Economic Forum (WEF): Measuring Stakeholder Capitalism report [29]	Generic
Coalition for Inclusive Capitalism: Embankment Project for Inclusive Capitalism (EPIC) report [22]	Generic
World Business Council for Sustainable Development (WBCSD): The Reporting Exchange [30]	Generic
Value Reporting Foundation: Sustainability Accounting Standards Board (SASB) standards [20]	Agricultural products; food retailers and distributors; meat, poultry & dairy; non-alcoholic beverages; processed foods; restaurants; alcoholic beverages
Access To Nutrition Initiative (ATNI): Global Index [21]	Food and beverage manufacturers
Access To Nutrition Initiative (ATNI): UK Retailer Index [21]	Food retail
The Food Foundation – Plating Up Progress (PUP) [31]	Supermarkets; contract caterers and food services; casual dining and restaurant chains; quick service restaurants; wholesalers
World Benchmarking Alliance (WBA) – Food and Agriculture Benchmark [18]	Agricultural inputs; animal proteins; food and beverage manufacturers/processors; food retailers; restaurant and food service

^a^We excluded frameworks that exclusively evaluated environmental sustainability or governance practices, provided only “guiding principles” for responsible investment, or were proprietary or required a purchased license.

^b^If the framework was designed to be universally applicable to businesses across sectors, we classify this as “generic”. If the framework is tailored to specific sectors, we note which sub-sectors of the food and beverage sector are covered.

One critical gap in ESG + Nutrition investing is an objective, accepted, evidence-based definition of healthfulness of diverse food and beverage products. We believe this will require a valid, flexible nutrient profiling system (NPS)—an algorithm incorporating multiple key nutritional characteristics—that can uniformly and accurately assess diverse food products, beverages, and mixed dishes or meals. Among current ESG frameworks, only ATNI includes an NPS-based measure of product portfolio healthfulness, based on the Health Star Rating [24], with further calls to action to use an NPS in the recent Nutrition For Growth (N4G) Investor Pledge [23]. While use of an NPS is a strength, the endorsed Health Star Rating has important limitations: it scores a small number of nutrients and ingredients, scores foods per gram and is thus unduly influenced by water weight, and uses different scoring principles and algorithms for subjectively grouped food categories [24]. Other popular NPS such as the U.K. traffic light labeling system and Pan American Health Organization (PAHO) Nutrient Profile Model additionally prioritize attributes like total fat, with outdated evidence for health impact, while not incorporating updated evidence on relevant food attributes [25, 26]. The new Food Compass appears to be a superior NPS for ESG + Nutrition, assessing multiple nutritional and processing characteristics per calorie of food, incorporating the best current scientific evidence on diverse attributes, and rating all foods, beverages, and mixed meals with a single objective scoring system [27]. With these strengths, the Food Compass can be used as the foundation for a range of ESG + Nutrition metrics, as discussed below.

## A proposed framework for objective, valid, and practical ESG + Nutrition metrics

**Fig. 2:**
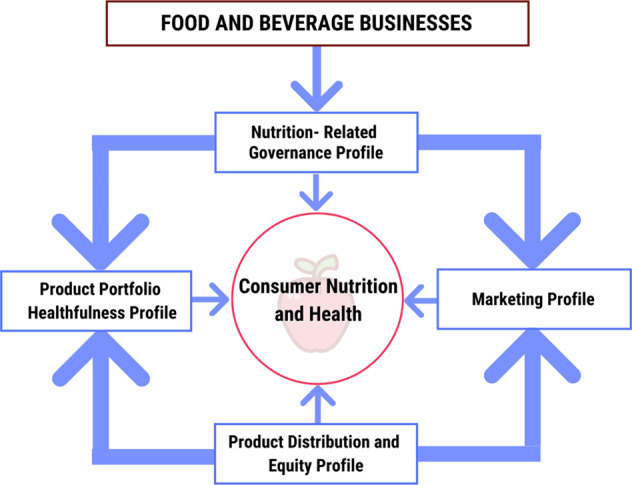
A proposed conceptual framework of ESG + Nutrition investing profiles to characterize how consumer-facing food and beverages businesses impact consumer nutrition and health. Consumer-facing food and beverage businesses include food and beverage manufacturers, food retailers, quick service and dine-in restaurants, and contract catering and food service. These businesses’ governance practices directly affect their product portfolio, marketing, and distribution and equity profiles, all of which influence consumer nutrition and health.

We propose a framework to develop objective, valid, and practical ESG + Nutrition metrics across four domains: (1) healthfulness of product portfolios, (2) equitability (affordability, accessibility) of product distribution across diverse populations, (3) marketing strategies and practices, and (4) corporate governance and other strategies related to nutrition (Fig. 2, Table 2). We believe the initial focus should be on consumer-facing food and beverage businesses – food and beverage manufacturers, food retailers, quick service and dine-in restaurants, and contract catering and food service – and later consider other food-related sectors, e.g., agricultural production, supply chains.

**Table 2:** Proposed new ESG + Nutrition metrics^a^ to evaluate the impact of consumer-facing food and beverage sector businesses on consumers’ nutrition and health.

Domains^b^ and affiliated metrics	Potential metric definitions
Product portfolio Profile	
Healthfulness	overall sales-weighted measure of healthfulness of products, based on either a continuous score or proportion meeting/not meeting certain threshold, using a validated NPS^c^
Product distribution and equity Profile	
Affordability of healthful foods	within company: ratio of price of 1 serving of healthful product to 1 serving unhealthful products (stratified by calorie bands), as defined by NPS
	between company: average price of 1 serving of healthful products, as defined by NPS (stratified by calorie bands) across companies
Financial accessibility of healthful foods	across country percentage of sales (PPP-adjusted) in top income quintile, bottom income quintile between countries - stratified by categories (quintiles) of healthfulness, as defined by NPS
	within country: percentage of sales based on relative income (comparison of top vs. bottom quintile) in income-matched units - stratified by categories (quintiles) of healthfulness, as defined by NPS
Geographic accessibility of healthful foods	within country: percentage of sales by Census track (or equivalent geographic indicator) - stratified by categories (quintiles) of healthfulness, as defined by NPS
Marketing Profile	
Marketing of healthful foods	within company: ratio of percentage marketing spending towards healthful products to unhealthful products, as defined by NPS - Stratified by race/ethnicity customer segment between company: percentage of marketing spending towards healthful products, as defined by NPS, compared to competitors - Stratified by race/ethnicity customer segment
Health claims	percentage of total product portfolio with marketing or package label health claims aligned with latest scientific evidence regarding health benefits of ingredients or nutrients
Responsible marketing policies	adherence score to ICC articles or equivalent for responsible marketing of food and beverages
	adherence score to CFBAI standards or equivalent for responsible marketing to children
	adherence score to International Code of Marketing of BMS or national equivalent for responsible marketing to mothers
Nutrition-related governance Profile	
Corporate nutrition strategy	meet or exceed goals related to product portfolio profile, product distribution and equity profile, and marketing profile metrics
Nutrition education	commitment to (and/or performance on) educating the general public about nutrition and health, in line with the latest evidence
Innovation, research and development	number and percentage of new healthful products launched, as defined by NPS

*BMS* Breastmilk substitutes; *CFBAI* Children’s Food and Beverage Advertising Initiative; *ESG* Environmental, social, and governance; *ICC* International Chamber of Commerce; *NPS* Nutrient profiling system; *PPP* Purchasing power parity.

^a^Metrics proposed encompass all or the majority of the following attributes identified for strong ESG metrics: (1) measure outputs of the company, including sales or performance with direct impacts on key consumers or community stakeholders; (2) are quantitative in resolution; (3) require minimal data assumptions for measurement; (4) utilize data that is publicly or privately available without substantial back-end effort for aggregation; (5) do not require the reporting entity to take subjective decisions on how to report; (6) measure the appropriate scope, with the flexibility to compare across companies, geographies and sub-sectors; (7) measure the intended construct; and (8) are adaptable as the science evolves over time.

^b^Consumer-facing food and beverage business can contribute to nutrition and health through four broad domains: (1) through the types of products they sell (e.g., their product portfolios); (2) through the equitability of the distribution (affordability, accessibility) of these products; (3) through their marketing strategies and practices around these products; and (4) through their larger governance and other strategies related to nutrition.

^c^Product healthfulness must be measured by an objective, validated NPS. In addition, the use of categorical cut-points versus a standardized continuous scale for healthfulness must be decided upon *apriori* to evaluate the relative healthfulness of individual food and beverage products, and thereby assess the overall healthfulness of a business’ product portfolio.

As described above, the health impacts of products should be quantified by a validated NPS, that scores foods, beverages, and meals on a range of protective and risk factor nutrients, ingredients, bio-actives, additives, and processing attributes. Such an NPS should be validated against clinical health outcomes and be amenable to updates over time based on scientific advances. To avoid subjectivity, the ideal NPS should also utilize the same scoring attributes and algorithm across different products. And, the NPS algorithm should be transparent and publicly available. We believe the Food Compass, which meets each of these criteria [27], should be considered as a crucial quantitative measure that allows measurement of an entire company’s product offerings, comparable over time, within and between companies.

We propose the equitable distribution of a company’s products as a second measure of ESG + Nutrition. Except for ATNI and WBA, existing ESG frameworks on nutrition fail to address this important dimension. Equitable distribution should incorporate the relative affordability and accessibility of healthful products, evaluated through an equity lens across racial, ethnic, geographic, and socioeconomic levels.

We believe ESG + Nutrition should also assess marketing strategies, including spending on different products and population targets, adherence to international standards, and message alignment with the latest science. Existing metrics focus only on company commitments or major legal infractions related to labeling and marketing statutes. Instead, quantitative measure of marketing strategy must be considered in relation to both the healthfulness and equitable targeting of products across different customer segments. Adherence to national and international marketing standards provides a performance measure of commitment to responsible marketing. An audit of health-related statements and claims (e.g., on product labels, advertisements, etc.) can provide further important data on whether a company’s nutrition and health messaging is evidence-based or potentially misleading to consumers without meeting the high bar of legal infraction.

A company’s governance strategies toward improving nutrition are a relevant, fourth dimension of ESG + Nutrition, evaluated alongside benchmarks of progress towards those aims to facilitate accountability. This can build upon existing ESG metrics for strategic targets and commitments around food product formulations and social responsibility, with further, novel aspects such as product innovations and nutrition education campaigns.

## ESG + Nutrition and financial performance

A successful ESG + Nutrition framework must track with and predict not only nutrition and health goals but also corporate financial performance. Some current ESG metrics, such as SASB’s standards, are created using an iterative process including research working group deliberations and public comment periods that consider perceived conceptual relationships of ESG topics with financial performance and social/environmental impact [28]. However, the relationship of most individual ESG metrics with these outcomes have yet to be validated. For long-term viability, proposed ESG + Nutrition metrics must be tested for dual materiality - enterprise value creation and human health.

## ESG + Nutrition accountability

Investor demand for ESG-conscious business practices is rapidly rising, but without mandatory ESG reporting structures or oversight. In this setting, food sector businesses generally report voluntarily on ESG performance, without consistency in which frameworks are used for reporting, which selected metrics are disclosed, and to whom disclosures are reported. Thus, businesses may select and report only on metrics that highlight positive practices. For example, based on our review of 2020 sustainability reports for global restaurant chains (McDonald’s, Starbucks, others) and retailers (Walmart, Kroger, others), some food sector businesses are developing their own metrics to fit business and philanthropic objectives, while others provide broad position statements on commitments to the environment, society, and good governance without concrete metric disclosures. Similarly, except for recommending NPS-based sales and portfolio reporting, the N4G Investor Pledge places the onus on each company to decide, develop, and disclose on SMART targets and metrics [23]. In sum, objectivity and accountability in ESG reporting across the food sector are suboptimal. To address this, ideally ESG + Nutrition metrics should be adopted by an independent and objective reporting body that promotes, oversees, and disseminates the findings.

## Next directions

We believe ESG + Nutrition has tremendous potential for shifting financial incentives toward more healthful food products that advance well-being, nutrition security, and health equity. This next stage in the investment landscape requires refinement, testing, and scaling of the proposed metrics in order to:

- Identify and validate an NPS as an objective, discriminatory measure of product healthfulness across food, beverage, and mixed meal portfolios that penalizes unhealthful products and provides credit for improvement to product portfolios.
- Identify optimal data sources to assess equity of portfolio distribution across population segments, marketing investments and practices, and nutrition-related governance.
- Demonstrate relationships with corporate financial performance with human health.
- Support decision-making of investors and food sector businesses; and understand the recursive relationships between investors, business strategies and outputs, and consumer demand to fuel growth of ESG + Nutrition.
- Assess opportunities for synergy with other ESG domains (e.g., environmental sustainability).
- Identify and regularly update findings on impacts of ESG + Nutrition metrics on product portfolios, distribution, marketing, and nutrition-related governance.
- Consider and address the need for an independent oversight body to harmonize data collection, analysis, and reporting by companies to support the rapidly evolving ESG regulatory environment.

## Conclusions

Momentum around ESG investing and the growing recognition of both risks and opportunities in the food sector have attracted the attention of multiple stakeholders — ESG-minded investors, private food sector businesses themselves, and academic and other non-profit organizations aiming to develop meaningful standards to drive ethical investing. At N4G, a landmark pledge by 53 institutional investors representing $12.4 trillion in assets under management [23] has called on food and beverage companies to report on the healthfulness of their product portfolios and sales; use an NPS to define healthy products; and adopt SMART governance, strategy, lobbying, and transparency commitments. To meet the needs of this growing demand by investors and companies, we believe the time is ripe for harmonized, evidence-based ESG-Nutrition metrics to guide food sector practices toward nutrition, health, and equity globally.
